# MAFLD and NAFLD in the prediction of incident chronic kidney disease

**DOI:** 10.1038/s41598-023-27762-6

**Published:** 2023-01-31

**Authors:** So Yoon Kwon, Jiyun Park, So Hee Park, You-Bin Lee, Gyuri Kim, Kyu Yeon Hur, Janghyun Koh, Jae Hwan Jee, Jae Hyeon Kim, Mira Kang, Sang-Man Jin

**Affiliations:** 1 Division of Endocrinology and Metabolism, Department of Medicine, Samsung Medical Center, Sungkyunkwan University School of Medicine, 81 Irwon‑ro, Gangnam‑gu, Seoul, 06351 Republic of Korea; 2 Division of Endocrine and Metabolism, Department of Internal Medicine, CHA Bundang Medical Center, CHA University School of Medicine, 59 Yatap-ro, Bundang-gu, Seongnam, Gyeonggi-do 14396 Republic of Korea; 3 Department of Health Promotion Center, Center for Health Promotion, Samsung Medical Center, Sungkyunkwan University School of Medicine, 81, Irwon-ro, Gangnam-gu, Seoul, 06351 Republic of Korea; 4 Department of Digital Health, SAIHST, Sungkyunkwan University, Seoul, Republic of Korea

**Keywords:** Hepatology, Endocrine system and metabolic diseases

## Abstract

Whether metabolic dysfunction-associated fatty liver disease (MAFLD) can replace nonalcoholic fatty liver disease (NAFLD) is under debate. This study evaluated which definition better predicted incident chronic kidney disease (CKD). This was a 5.3-year (range, 2.8–8.3) retrospective cohort study of 21,713 adults who underwent at least two serial health examinations. Cox analyses were used to compare the risk of incident CKD among non-fatty liver disease (FLD) without metabolic dysregulation (MD; reference), non-FLD with MD, MAFLD-only, NAFLD-only, or both-FLD groups. Non-FLD with MD group (hazard ratio [HR] 1.23, 95% confidence interval [CI] 1.00–1.53), both-FLD group (HR 1.50, 95% CI 1.19–1.89), and MAFLD-only group (HR 1.97, 95% CI 1.49–2.60), but not NAFLD-only group (HR 1.06, 95% CI 0.63–1.79) demonstrated an increased risk of CKD. The increased risk of CKD was significant in MAFLD subgroups with overweight/obesity (HR 2.94, 95% CI 1.91–4.55), diabetes (HR 2.20, 95% CI 1.67–2.90), MD only (HR 1.50, 95% CI 1.19–1.89), excessive alcohol consumption (HR 2.71, 95% CI 2.11–3.47), and viral hepatitis (HR 2.38, 95% CI 1.48–3.84). The switch from NAFLD to MAFLD criteria may identify a greater number of individuals at CKD risk. The association was also significant in MAFLD patients with excessive alcohol consumption or viral hepatitis.

## Introduction

Non-alcoholic fatty liver disease (NAFLD) is a global health issue that affects about a quarter of the world’s population^1^. Numerous studies have shown unequivocally that NAFLD is a hepatic manifestation of systemic metabolic diseases^2,3^. However, criteria used to diagnose NAFLD do not allow for the presence of excessive alcohol intake or viral hepatitis even in the presence of metabolic dysfunction that may have contributed to the presence of steatosis^4,5^. In this context, an international consensus panel proposed a new nomenclature for NAFLD, namely ‘metabolic dysfunction–associated fatty liver disease’ (MAFLD)^6^. This new nomenclature has inclusion criteria in contrast to NAFLD and is defined by evidence of fatty liver disease (FLD) in addition to one of the following three features: overweight/obese, type 2 diabetes, or metabolic dysregulation. By definition, MAFLD includes those with excessive alcohol intake or other concomitant liver diseases while excluding those who do not meet the metabolic dysregulation requirements.

Several studies have been conducted to determine whether MAFLD or NAFLD classifications better predict mortality or extrahepatic metabolic complications such as chronic kidney disease (CKD). However, whether MAFLD can successfully replace NAFLD is still under debate. While three cross-sectional studies showed that MAFLD was better at identifying patients with significant hepatic fibrosis or CKD^7–9^, one longitudinal retrospective study showed that MAFLD was not associated with a higher incidence of diabetes, CKD, or cardiovascular disease (CVD) compared with NAFLD^10^. Furthermore, two longitudinal studies with mortality as an outcome reported contradictory results^11,12^.

However, whether the risk of extrahepatic outcomes such as CKD is significantly increased in FLD patients with metabolic dysregulation, excessive drinking, or other liver diseases compared to patients without such conditions has yet to be elucidated in a large-scale longitudinal cohort study. We therefore evaluated the associated risks for CKD events according to NAFLD and MAFLD criteria and assessed which definition was better at identifying risk of incident CKD based on a longitudinal analysis of a community-based cohort.

## Methods

### Study design

This longitudinal retrospective cohort study included subjects aged 20 years and older who underwent at least two comprehensive health examinations. The first health examination was conducted between January 2001 and July 2016, and the last health examination was conducted up to December 2020 at the Health Promotion Center at Samsung Medical Center (SMC, Seoul, Republic of Korea). The study population consisted of employees of various institutions and companies required to undergo a comprehensive health checkup annually or biennially by the Industrial Safety and Health Law of the Republic of Korea. All subjects underwent abdominal ultrasound at the first health examination to assess the presence and severity of hepatic steatosis. The study protocol was approved by the Institutional Review Board (IRB) of Samsung Medical Center (SMC; Seoul, Republic of Korea) (no. 2021–05-025), and the requirement for informed consent was waived by the IRB because the study information was de-identified. The protocol for the study adhered to the guidelines of the Declaration of Helsinki.

### Study population

**Figure 1:**
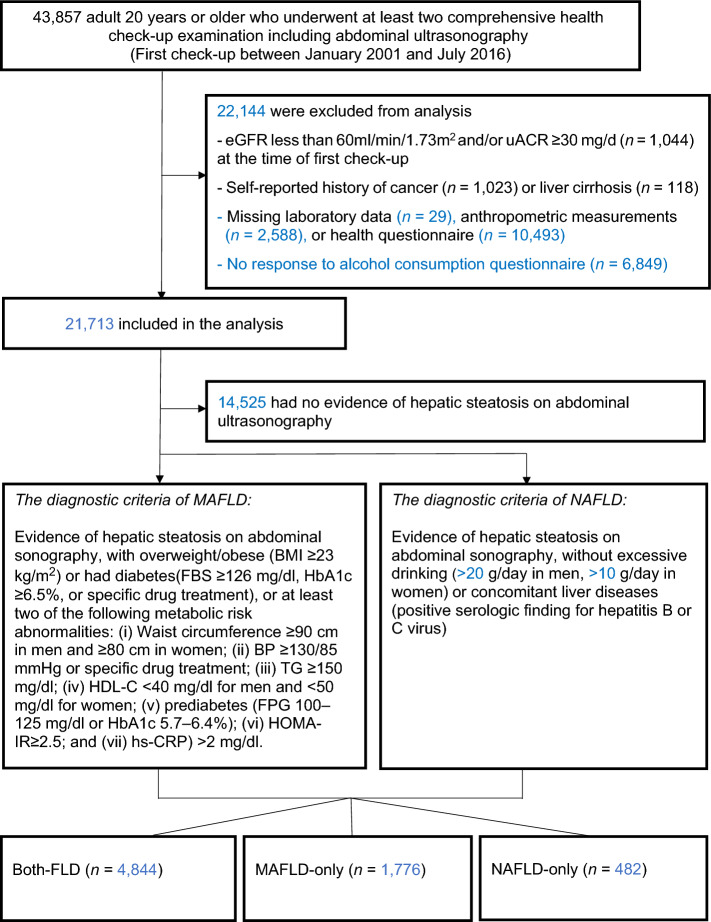
Flow diagram of the study population.

In total, 43,857 subjects who had at least two serial health examinations and underwent abdominal ultrasonography at the first health examination were identified. Subjects with an estimated glomerular filtration rate (eGFR) less than 60 mL/min/1.73 m^2^ as calculated by the Chronic Kidney Disease Epidemiology Collaboration (CKD-EPI) formula^13^ and/or urine albumin/creatinine ratio (uACR) greater than or equal to 30 mg/g at the time of first health examination were excluded from this cohort (*n* = 1,044). We also excluded subjects with a history of malignant diseases (*n* = 1,023) or history of liver cirrhosis (*n* = 118). We further excluded subjects with missing laboratory data (*n* = 29), anthropometric measurements (*n* = 2,588), health questionnaires (*n* = 10,493), or questionnaires regarding daily alcohol consumption (*n* = 6,849), leaving a final study population of 21,713 for longitudinal analyses (Fig. 1).

### Measurements of clinical and laboratory data

During health examination, laboratory, anthropometric, and questionnaire-based data were collected. The self-administered questionnaire assessed prior medical history, surgical history, prescribed medications, daily alcohol consumption (g/day), smoking status, and exercise status. Excessive alcohol intake was defined as > 20 g/day in men and > 10 g/day in women. Subjects were categorized as never, former, or current smokers. Exercise status was classified into 0 days, 1–2 days, 3–4 days, or 5 days per week.

Body mass index (BMI) was calculated as body weight divided by height squared (kg/m^2^). Overweight and obesity were defined as BMI ≥ 23 kg/m^2^ and ≥ 25 kg/m^2^, respectively, according to Asian-specific criteria. CKD was defined as an eGFR less than 60 mL/min/1.73 m^2^ and/or urine albumin/creatinine ratio greater than or equal to 30 mg/g. Hepatitis B was defined as present if the subject was positive for hepatitis B virus surface antigen (HBsAg), while hepatitis C was diagnosed if the subject was hepatitis C antibody (Anti-HCV Ab) positive. Diabetes mellitus (DM) was defined as a fasting plasma glucose (FPG) ≥ 126 mg/dl, hemoglobin A1c (HbA1c) ≥ 6.5%, or use of specific drugs. Prediabetes was defined as a fasting blood glucose level of 100–125 mg/dl or HbA1c of 5.7–6.4% in subjects without a prior diagnosis of DM. Hypertension was identified as a systolic blood pressure (SBP) ≥ 140 mmHg, diastolic blood pressure (DBP) ≥ 90 mmHg, and/or taking antihypertensive medication. Homeostasis model assessment of insulin resistance score (HOMA-IR) was calculated as FPG (mg/dl) x fasting plasma insulin (µIU/ml)/405. Nonalcoholic fatty liver disease fibrosis score (NFS) was calculated according to the following formula: − 1.675 + 0.037 × age (years) + 0.094 × BMI (kg/m^2^) + 1.13 × impaired fasting glucose/DM (yes = 1, no = 0) + 0.99 × AST/ALT ratio − 0.013 × platelet count (× 10^9^/l) − 0.66 × albumin (g/dl)^14^.

### Definitions of MAFLD and NAFLD

The presence of hepatic steatosis was assessed by abdominal ultrasonography using standard criteria such as parenchymal brightness, liver-to-kidney contrast, deep beam attenuation, and bright vessel walls. If present, radiologists graded the severity of hepatic steatosis as mild, moderate, or severe^15,16^.

Abdominal ultrasonography was performed using LogiQ E9 (GE Healthcare, Milwaukee, WI, USA), iU22 xMatrix (Philips Medical Systems, Cleveland, OH, USA), or ACUSON Sequoia 512 instruments (Siemens, Issaquah, WA, USA).

NAFLD was defined when there was evidence of hepatic steatosis on ultrasonography without excessive alcohol intake or other concomitant liver diseases such as viral hepatitis. MAFLD was diagnosed when participants with hepatic steatosis on ultrasonography had at least one of the following three conditions: overweight/obese (BMI ≥ 23 kg/m^2^), presence of DM, or evidence of metabolic dysregulation (MD). MD was defined as at least two of the following: 1) waist circumference ≥ 90 cm in men and ≥ 80 cm in women; 2) BP ≥ 130/85 mmHg or treatment with antihypertensive drugs; 3) triglycerides (TG) ≥ 150 mg/dl or specific drug treatment; 4) high-density lipoprotein cholesterol (HDL-C) < 40 mg/dl for men and < 50 mg/dl for women; 5) prediabetes; 6) HOMA-IR ≥ 2.5; and/or 7) high-sensitivity C-reactive protein (hs-CRP) > 2 mg/dl.

We identified subjects without evidence of hepatic steatosis and split them into two groups according to their MD status (non-FLD without MD or non-FLD with MD). We classified subjects into the both-fatty liver disease (both-FLD) group if they met both MAFLD and NAFLD definitions. Subjects who met the definition of MAFLD but not NAFLD were assigned to the MAFLD-only group, whereas those with NAFLD but not MAFLD were assigned to the NAFLD-only group. MAFLD was further divided into three subgroups: 1) MAFLD with excessive alcohol consumption; 2) MAFLD with viral hepatitis; 3) MAFLD with MD only (provided that the subject did not drink excessively and did not have viral hepatitis; the same group as both-FLD group).

### Statistical analysis

Continuous variables are expressed as means ± standard deviation (SD), and categorical variables are presented as frequencies (%). We compared continuous baseline characteristics using one-way ANOVA and categorical baseline variables using the chi-square test. The Scheffe test was used for post-hoc analysis. Cox proportional hazards models were used to estimate the hazard ratios (HR) and 95% confidence intervals (CIs) for incident CKD. In multivariable-adjusted analyses, model 2 was adjusted for age and sex, while model 3 was additionally adjusted for eGFR, smoking status, excessive alcohol consumption, physical activity, NFS, and co-morbidities such as prediabetes, DM, hypertension, and a history of CVD.

All tests were two-sided and a *p*-value of < 0.05 (two-tailed) was considered statistically significant. Analyses were performed using SPSS version 22.0 (SPSS, Chicago, IL, USA) or R version 3.6.1 (R Foundation for Statistical Computing, Vienna, Austria).

## Results

### Baseline characteristics of participants

Of the 21,713 participants included in the analysis, 14,525 (66.9%) had no evidence of hepatic steatosis on baseline abdominal ultrasonography, of which 9,414 (43.4%) subjects did not have MD (non-FLD without MD) while 5,111 (23.5%) subjects had MD (non-FLD with MD). Among the subjects with FLD confirmed by ultrasound, 6,620 (30.5%) met the MAFLD definition and 5,326 (24.5%) met the NAFLD definition. A total of 4,844 (22.3%) subjects met the criteria of both-FLD, while 1,776 (8.2%) subjects were classified as MAFLD-only and 482 (2.2%) were diagnosed as NAFLD-only. The 86 (0.4%) subjects with FLD who did not have MD but had excessive alcohol intake or viral hepatitis were not classified into any of the aforementioned groups.

In Table 1, compared to the non-FLD without MD group and NAFLD-only group, subjects in the non-FLD with MD, MAFLD-only, and both-FLD groups were more likely to have higher BMI and waist circumference (WC) and more comorbidities. Laboratory data also demonstrated that subjects in non-FLD with MD, MAFLD-only, and both-FLD groups had a lower eGFR, more atherogenic lipid profile, and higher uACR, FPG, and HbA1c levels. HOMA-IR, hs-CRP, and NFS levels were lower in the non-FLD without MD and NAFLD-only groups. Mild FLD was more prevalent in the NAFLD-only group than the other groups based on ultrasound findings.

**Table 1:** Comparison of baseline characteristics in subjects.

Characteristics	Total N = 21,713	Non-FLD without MD n = 9,414 (43.4%)	Non-FLD with MD n = 5,111 (23.5%)	MAFLD-only n = 1,776 (8.2%)	NAFLD-only n = 482 (2.2%)	Both-FLD n = 4,844 (22.3%)	*P* value
Age, yrs	44 ± 10	42 ± 10	47 ± 9	47 ± 8	45 ± 9	46 ± 9	< 0.01
Male sex, *n* (%)	14,783 (68.08)	4,472 (47.50)	3,922 (76.74)	682 (94.99)	408 (76.55)	5,267 (89.24)	< 0.01
BMI, kg/m^2^	23.62 ± 3.05	21.42 ± 1.98	24.72 ± 2.37	26.38 ± 2.71	21.71 ± 1.10	25.93 ± 2.49	
Excess drinker, *n* (%)^†^	4,101 (18.89)	1,203 (12.78)	1,260 (24.65)	1,568 (88.29)	0 (0)	0 (0)	< 0.01
Hepatitis B, *n* (%)^†^	878 (4.04)	374 (3.97)	250 (4.89)	233 (13.13)	0 (0)	0 (0)	< 0.01
Hepatitis C, *n* (%)^†^	108 (0.50)	52 (0.55)	27 (0.53)	29 (1.63)	0 (0)	0 (0)	< 0.01
Current smoker, *n* (%)	5,991 (27.66)	1,887 (20.10)	1,549 (30.37)	816 (46.08)	121 (25.16)	1,574 (32.57)	< 0.01
Comorbidities, *n* (%)							< 0.01
Diabetes^†^	1,147 (5.28)	0 (0)	414 (8.10)	237 (13.34)	0 (0)	496 (10.24)	
Prediabetes^†^	5,239 (24.13)	856 (9.09)	1,812 (36.04)	728 (40.99)	89 (18.46)	1,708 (35.26)	
Hypertension^†^	7,145 (32.91)	4,854 (24.55)	2,605 (50.97)	971 (54.67)	66 (13.69)	2,316 (47.81)	
WC, cm							
In men	87.14 ± 7.40	81.31 ± 5.42	88.31 ± 6.17	92.07 ± 6.74	81.83 ± 4.11	90.77 ± 6.44	< 0.01
In women	74.96 ± 7.51	72.30 ± 5.71	80.82 ± 6.68	87.69 ± 7.00	73.85 ± 5.24	84.02 ± 7.48	< 0.01
BUN, mg/dl	23.62 ± 3.05	12.61 ± 3.18	13.43 ± 3.33	13.78 ± 3.22	13.13 ± 3.11	13.72 ± 3.14	< 0.01
Creatinine, mg/dl	0.91 ± 0.17	0.86 ± 0.17	0.94 ± 0.16	0.97 ± 0.13	0.92 ± 0.16	0.97 ± 0.15	< 0.01
eGFR, ml/min/1.73 m^2^	94.06 ± 13.89	97.42 ± 13.96	91.70 ± 13.43	91.65 ± 12.53	94.32 ± 12.91	90.88 ± 13.39	< 0.01
uACR, μg/mgCr	5.62 ± 4.91	5.09 ± 4.42	5.60 ± 4.98	6.45 ± 5.48	5.11 ± 4.40	6.16 ± 5.24	< 0.01
AST, IU/L	23.25 ± 16.41	20.78 ± 17.62	22.80 ± 12.62	29.20 ± 19.74	21.76 ± 7.38	26.32 ± 14.83	< 0.01
ALT, IU/L	25.33 ± 26.22	18.63 ± 28.49	24.05 ± 16.03	36.86 ± 28.22	24.29 ± 14.08	35.42 ± 25.87	< 0.01
FPG, mg/dl	92.87 ± 15.97	86.93 ± 7.61	96.18 ± 18.30	102.15 ± 22.59	89.90 ± 7.99	97.84 ± 18.59	< 0.01
HbA1c, %	5.38 ± 0.63	5.18 ± 0.34	5.48 ± 0.72	5.65 ± 0.86	5.28 ± 0.37	5.57 ± 0.75	< 0.01
HOMA-IR	1.87 ± 1.20	1.30 ± 0.67	1.97 ± 1.17	2.54 ± 1.40	1.48 ± 0.71	2.46 ± 1.40	< 0.01
TGs, mg/dl	119.10 ± 35.77	82.45 ± 35.77	129.08 ± 73.75	177.11 ± 112.19	109.90 ± 54.60	159.31 ± 88.64	< 0.01
HDL-C, mg/dl	56.63 ± 14.63	63.34 ± 14.55	53.89 ± 13.48	50.49 ± 12.03	56.88 ± 12.71	48.71 ± 10.61	< 0.01
Hs-CRP, mg/dl	0.12 ± 0.34	0.09 ± 0.20	0.15 ± 0.50	0.17 ± 0.41	0.10 ± 0.16	0.16 ± 0.35	< 0.01
NFS	-2.52 ± 1.09	-2.85 ± 1.01	-2.17 ± 1.08	-2.11 ± 1.05	-2.88 ± 1.05	-2.33 ± 1.08	< 0.01
US steatosis severity, *n* (%)							
Normal	14,525 (66.90)	9,414 (100)	5,111 (100)	0 (0)	0 (0)	0 (0)	
Mild	4,309 (19.85)	0 (0)	0 (0)	1087 (61.20)	381 (79.05)	2,765 (57.08)	
Moderate	2,621 (12.07)	0 (0)	0 (0)	634 (35.70)	97 (20.12)	1,880 (38.81)	
Severe	258 (1.19)	0 (0)	0 (0)	55 (3.10)	4 (0.83)	199 (4.11)	

Data are shown as mean ± standard deviations or frequency as appropriate. P-value was calculated using One-way ANOVA test for continuous variables and Chi-square test for categorical variables. The Scheffe test was used in post-hoc analysis.

*ALT* alanine aminotransferase, *AST* aspartate aminotransferase, *BMI* body mass index, *DBP* diastolic blood pressure, *eGFR* estimated glomerular filtration rate, *FPG* fasting plasma glucose, *FLD* fatty liver disease, *HbA1c* glycated hemoglobin A1c, *HBV* hepatitis B virus, *HCV* hepatitis C virus, *HDL-C* high-density lipoprotein cholesterol, *HOMA-IR* homeostasis model assessment of insulin resistance, *hs-CRP* high-sensitivity C-reactive protein, *LDL-C* low-density lipoprotein cholesterol, *MAFLD* metabolic dysfunction–associated fatty liver disease, *NAFLD* nonalcoholic fatty liver disease, *NFS* NAFLD fibrosis score, *SBP* systolic blood pressure, *TGs* triglycerides, *uACR* urinary albumin to creatinine ratio, *US* ultrasonography, *WC* waist circumference.

^†^Overweight/obesity: BMI of 23.0 or greater; hypertension: blood pressure greater than or equal to 130/85 mm Hg or specific drug treatment; diabetes: fasting blood glucose greater than or equal to 126 mg/dl, or HbA1c greater than or equal to 6.5% or specific drug treatment; prediabetes: fasting glucose 100 to 125 mg/dl or HbA1c 5.7% to 6.4% in participants without a prior diabetes diagnosis; excess drinker was defined as more than 20 g daily of alcohol consumption in men and more than 10 g in women; Hepatitis B was defined as positive HBsAg; Hepatitis C was defined as positive anti-HCV antibody.

### Association of MAFLD and NAFLD status with incident CKD

**Figure 2:**
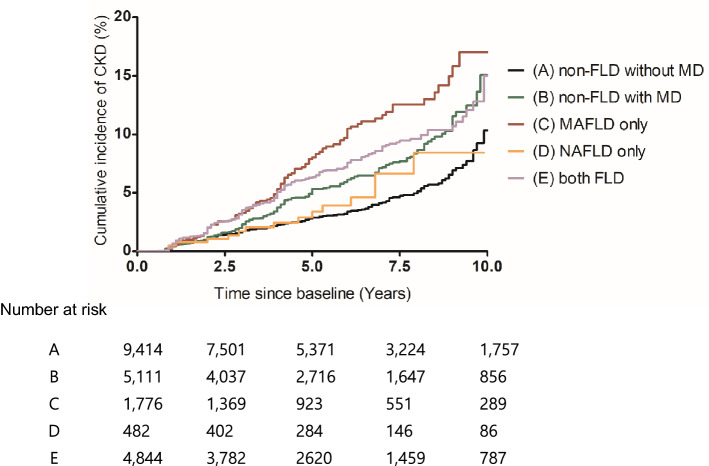
Cumulative CKD incidence over 10 years by Kaplan–Meier methods according to presence and combination of MAFLD and NAFLD-non-FLD with or without MD, MAFLD only, NAFLD only, or both FLD.

During the follow-up period (median 5.3 years, interquartile range: 2.8–8.3 years), CKD developed in 912 participants (4.2%). The cumulative CKD incidence of each group over 10 years is shown in Fig. 2. Compared with non-FLD, FLD subjects had increased risks of incident CKD (HR 1.34; 95% CI, 1.14–1.56) after adjusting for known risk factors (Table 2). Association of non-FLD with or without MD, MAFLD-only, NAFLD-only, or both-FLD and incidence of CKD are summarized in Table 2. After adjusting for known risk factors, both-FLD and MAFLD-only groups had a 1.50 (95% CI, 1.19–1.89) and 1.97 (95% CI, 1.49–2.60) times higher CKD risk than the non-FLD without MD group. Additionally, even in participants without hepatic steatosis, subjects with MD had a higher incidence of CKD (HR 1.23; 95% CI 1.00–1.53). However, the NAFLD-only group did not have an increased risk of CKD (HR 1.06, 95% CI, 0.63–1.79).

**Table 2:** Associations of MAFLD and NAFLD status with incident CKD.

	Cases, n	Event, n	Hazard ratio (95% CI)					
			Model 1^†^ (95%CI)	*P*	Model 2^‡^ (95% CI)	*P*	Model 3^§^ (95%CI)	*P*
Non-FLD	14,525	498	Ref		Ref		Ref	
FLD	7,188	414	1.74 (1.53–1.99)	< 0.01	1.67 (1.45–1.91)	< 0.01	1.34 (1.14–1.56)	< 0.01
Non-FLD without MD^¶^	9,414	259	Ref		Ref		Ref	
Non-FLD with MD^¶^	5,111	239	1.78 (1.49–2.12)	< 0.01	1.57 (1.31–1.89)	< 0.01	1.23 (1.00–1.53)	0.05
Both-FLD	4,844	270	2.13 (1.80–2.52)	< 0.01	2.01 (1.67–2.41)	< 0.01	1.50 (1.19–1.89)	< 0.01
MAFLD-only	1,776	125	2.77 (2.24–3.43)	< 0.01	2.72 (2.16–3.41)	< 0.01	1.97 (1.49–2.60)	< 0.01
NAFLD-only	482	15	1.12 (0.67–1.89)	0.66	1.06 (0.63–1.78)	0.84	1.06 (0.63–1.79)	0.82

*CI* confidence interval, *CKD* chronic kidney disease, *FLD* fatty liver disease, *MAFLD* metabolic dysfunction–associated fatty liver disease, *MD* metabolic dysregulation, *NAFLD* nonalcoholic fatty liver disease.

^†^Model 1 was crude.

^**‡**^Model 2 was adjusted for age and sex.

^**§**^Model 3 was adjusted for age, sex, estimated glomerular filtration rate, smoking, physical activity, prediabetes, diabetes, hypertension, cardiovascular disease, NAFLD fibrosis score, body mass index.

^¶^Metabolic dysregulation; subjects with overweight/obese (body mass index ≥ 23 kg/m^2^) or had diabetes (fasting plasma glucose [FPG] ≥ 126 mg/dl, hemoglobin A1c [HbA1c] ≥ 6.5%, or specific drug treatment), or at least two of the following metabolic risk abnormalities: (i) waist circumference ≥ 90 cm in men and ≥ 80 cm in women; (ii) blood pressure ≥ 130/85 mmHg or specific drug treatment; (iii) triglycerides ≥ 150 mg/dl; (iv) HDL-C < 40 mg/dl for men and < 50 mg/dl for women; (v) prediabetes (FPG 100–125 mg/dl or HbA1c 5.7–6.4%); (vi) homeostasis model assessment of insulin resistance ≥ 2.5; and (vii) high-sensitivity C-reactive protein > 2 mg/dl.

### Association between MAFLD and NAFLD status and incident CKD according to BMI category and presence of DM

Subgroup analyses were performed to determine the effect of overweight/obesity or diabetes, which are diagnostic criteria for MAFLD, on CKD development (Table 3). When categorizing participants as overweight/obese (BMI ≥ 23 kg/m^2^) or lean (BMI < 23 kg/m^2^), overweight/obese subjects with FLD were found to have a higher risk of CKD, regardless of their MAFLD or NAFLD status (HR 2.94; 95% CI, 1.91–4.55; HR 1.61; 95% CI, 1.32–1.96, respectively). Increased risk of incident CKD was not observed in NAFLD subjects with a BMI < 23 kg/m^2^.

**Table 3:** Associations between BMI and DM and the incident CKD in subjects with MAFLD or NAFLD.

	Cases, n	Event, n	Hazard ratio (95% CI)					
			Model 1^†^ (95%CI)	*P*	Model 2^‡^ (95%CI)	*P*	Model 3^§^ (95%CI)	*P*
Non-FLD with BMI < 23 kg/m^2^	8,138	244	Ref		Ref		Ref	
Non-FLD with BMI ≥ 23 kg/m^2^	6,387	254	1.36 (1.14–1.62)	< 0.01	1.31 (1.09–1.57)	< 0.01	1.21 (1.00–1.46)	0.04
MAFLD with BMI < 23 kg/m^2^	404	22	1.90 (1.23–2.94)	< 0.01	1.55 (0.10–2.41)	0.05	2.14 (1.15–3.97)	0.02
MAFLD with BMI ≥ 23 kg/m^2^	6,216	373	2.11 (1.79–2.48)	< 0.01	2.05 (1.72–2.45)	< 0.01	2.94 (1.91–4.55)	< 0.01
NAFLD with BMI < 23 kg/m^2^	786	29	1.22 (0.83–1.80)	0.30	1.11 (0.75–1.64)	0.60	1.00 (0.67–1.48)	1.00
NAFLD with BMI ≥ 23 kg/m^2^	4,540	256	1.97 (1.65–2.34)	< 0.01	1.95 (1.61–2.36)	< 0.01	1.61 (1.32–1.96)	< 0.01
Non-FLD without DM	14,111	470	Ref		Ref		Ref	
Non-FLD with DM	414	28	2.35 (1.61–3.45)	< 0.01	1.65 (1.12–2.44)	0.01	1.59 (1.08–2.36)	0.02
MAFLD without DM	5,887	326	1.72 (1.50–1.98)	< 0.01	1.67 (1.44–1.94)	< 0.01	1.39 (1.18–1.65)	< 0.01
MAFLD with DM	733	69	3.21 (2.50–4.14)	< 0.01	2.74 (2.11–3.55)	< 0.01	2.20 (1.67–2.90)	< 0.01
NAFLD without DM	4,830	239	1.51 (1.29–1.77)	< 0.01	1.47 (1.25–1.73)	< 0.01	1.25 (1.05–1.49)	0.01
NAFLD with DM	496	46	3.34 (2.47–4.53)	< 0.01	2.76 (2.03–3.76)	< 0.01	2.20 (1.60–3.04)	< 0.01

*BMI* body mass index, *CI* confidence interval, *CKD* chronic kidney disease, *DM* diabetes mellitus, *MD* metabolic dysregulation, *MAFLD* metabolic dysfunction–associated fatty liver disease, *NAFLD* nonalcoholic fatty liver disease.

The MAFLD group consists of both-FLD (n = 4,844; same as MAFLD with MD only group) and MAFLD-only (n = 1,776) groups, and the NAFLD group consists of both-FLD (n = 4,844) and NAFLD-only (n = 482) groups.

^†^Model 1 was crude.

^**‡**^Model 2 was adjusted for age and sex.

^**§**^Model 3 was adjusted for age, sex, estimated glomerular filtration rate, smoking, physical activity, prediabetes, diabetes, hypertension, cardiovascular disease, NAFLD fibrosis score, body mass index.

In both MAFLD and NAFLD groups, subjects with diabetes had a significantly higher risk of CKD (HR 2.20; 95% CI, 1.67–2.90; HR 2.20; 95% CI, 1.60–3.04, respectively). MAFLD or NAFLD subjects without diabetes still demonstrated an increased risk of incident CKD (HR 1.39; 95% CI, 1.18–1.65; HR 1.25; 95% CI, 1.05–1.49, respectively).

### Association between MAFLD and NAFLD status and incident CKD according to alcohol intake and viral hepatitis

Since MAFLD, in contrast to NAFLD, does not exclude patients with excessive alcohol consumption or viral hepatitis, we performed a subgroup analysis of the above groups (Table 4). A higher risk of incident CKD was observed among subjects with MAFLD or NAFLD (HR 1.60; 95% CI, 1.28–2.00; HR 1.43; 95% CI, 1.14–1.79, respectively). MAFLD with MD only (the same group as both-FLD group), MAFLD with excessive alcohol intake, or MAFLD with viral hepatitis groups had an increased risk of incident CKD (HR 1.50; 95% CI, 1.19–1.89; HR 2.71; 95% CI, 2.11–3.47, HR 2.38; 95% CI 1.48–3.84, respectively).

**Table 4:** Associations between alcohol, viral hepatitis and the incident CKD in subjects with MAFLD or NAFLD.

	Cases, n	Event, n	Hazard ratio (95% CI)					
			Model 1^†^ (95%CI)	*P*	Model 2^‡^ (95% CI)	*P*	Model 3^§^ (95%CI)	*P*
Non-FLD without MD	9,414	259	Ref		Ref		Ref	
Non-FLD with MD	5,111	239	1.78 (1.49–2.12)	< 0.01	1.57 (1.31–1.89)	< 0.01	1.23 (1.00–1.53)	0.05
MAFLD	6,620	395	2.30 (1.96–2.69)	< 0.01	2.16 (1.82–2.57)	< 0.01	1.60 (1.28–2.00)	< 0.01
With MD only^¶^	4,844	270	2.13 (1.80–2.52)	< 0.01	2.01 (1.67–2.41)	< 0.01	1.50 (1.19–1.89)	< 0.01
With excessive alcohol intake^#^	1,568	111	2.82 (2.26–3.53)	< 0.01	2.81 (2.21–3.58)	< 0.01	2.71 (2.11–3.47)	< 0.01
With viral hepatitis	263	19	2.63 (1.65–4.19)	< 0.01	2.49 (1.55–4/00)	< 0.01	2.38 (1.48–3.84)	< 0.01
NAFLD	5,326	285	2.03 (1.72–2.40)	< 0.01	1.93 (1.61–2.31)	< 0.01	1.43 (1.14–1.79)	< 0.01

The MAFLD group consists of both-FLD (n = 4,844; same as MAFLD with MD only group) and MAFLD-only (n = 1,776) groups, and the NAFLD group consists of both-FLD (n = 4,844) and NAFLD only (n = 482) groups.

*CI* confidence interval, *CKD* chronic kidney disease, *MAFLD* metabolic dysfunction–associated fatty liver disease, *NAFLD* nonalcoholic fatty liver disease.

^†^Model 1 was crude.

^**‡**^Model 2 was adjusted for age and sex.

^**§**^Model 3 was adjusted for age, sex, estimated glomerular filtration rate, smoking, physical activity, prediabetes, diabetes, hypertension, cardiovascular disease, NAFLD fibrosis score, body mass index.

^¶^Subjects with MAFLD who did not drink excessively and did not have viral hepatitis.

^#^Excess consumption of alcohol was defined as more than 20 g daily of alcohol consumption in men and more than 10 g in women.

### Risk of incident CKD according to the presence of FLD and metabolic dysfunction in subjects with excessive alcohol consumption or viral hepatitis

To determine the relative contribution of FLD and metabolic dysfunction to the significant association between MAFLD and CKD in subjects with excessive alcohol consumption or viral hepatitis, we compared the risk of incident CKD according to the presence of FLD and metabolic dysfunction in these subjects. Subjects with excessive alcohol intake alone in the absence of FLD did not significantly increase the risk of incident CKD (HR 1.37; 95% CI, 0.94–2.01). However, the risk of CKD was significantly increased in non-FLD subjects with excessive alcohol consumption and MD (HR 1.78; 95% CI, 1.28–2.48). A more prominent increase in the risk of CKD was demonstrated when excessive alcohol consumption and MD were combined with FLD (HR 3.10; 95% CI, 2.34–4.10; supplementary Table S1).

A similar trend was observed in subjects with viral hepatitis (Supplementary Table S2). The presence of viral hepatitis alone did not significantly increase the risk of CKD (HR 1.06; 95% CI, 0.59–1.89). The risk of CKD was significantly elevated when both viral hepatitis and MD were present (HR 2.16; 95% CI, 1.26–3.71), and a more prominent increase in such risk was documented when FLD was also present (HR 3.10; 95% CI, 1.81–5.29).

## Discussion

In this sizable retrospective cohort analysis, MAFLD with or without overlapping NAFLD was independently associated with an increased risk of CKD after adjusting for known risk factors for CKD. Subjects with NAFLD but not MAFLD did not have an increased risk of developing CKD. In subgroup analyses, the incidence of CKD was increased in lean MAFLD but not in lean NAFLD subjects. MAFLD subjects with excessive alcohol intake had a higher risk of incident CKD than MAFLD subjects with MD only, with significant interaction effects among excessive alcohol intake, MD, and FLD on the risk of CKD. A similar trend was also observed in MAFLD subjects with viral hepatitis. Since the definition of MAFLD was not established on evidence of metabolic complications such as CVD, CKD, and DM^6^, the results of this study can support the validity of the MAFLD definition.

The increase in risk of CKD in the MAFLD-only group but not in the NAFLD-only group supports the idea that the diagnosis of MAFLD may identify those with increased risk of CKD who might have been falsely reassured when considered to have NAFLD, excluding patients with viral hepatitis or alcoholism based on the assumption that hepatic steatosis develops independently in these conditions^17^. The results of this study are consistent with a recent cross-sectional study^8^ and a recent retrospective longitudinal study based on a national health insurance database, although the latter study defined FLD based on a fatty liver index higher than 30 without abdominal ultrasonographic evaluation, and HOMA-IR and hs-CRP levels were not available^8,18^. A recent longitudinal retrospective study in China indicated that while switching from NAFLD to MAFLD could identify FLD patients with excessive alcohol consumption and HBV infection, it had little effect on the associations with CKD or CVD^10^. However, the latter study did not focus on the comparison between those who met the criteria for either MAFLD or NAFLD but not the other, for whom a change in FLD definition would be most important^10^. In the current study, the HR for the risk of CKD in MAFLD with excessive alcohol intake (2.71, 95% CI 2.11–3.47) even exceeded that of MAFLD without excessive alcohol intake or viral hepatitis (1.50, 95% CI 1.19–1.89), which is the same group as NAFLD with MD (both-FLD group), supporting the hypothesis that switching from NAFLD to MAFLD would be better for detection of populations at risk of CKD (Table 4).

Several plausible pathophysiologic mechanisms have been suggested to explain the well-known association between NAFLD and CKD^19–26^. NAFLD may contribute to CKD development by rennin-angiotensin system activation, inflammatory factors, or metabolic factors such as abdominal obesity, insulin resistance, lipogenesis, and hyperglycemia^24,27,28^. The pathophysiological link between MAFLD and CKD may be similar to that between NAFLD and CKD^29^. Metabolic dysfunction may explain the association between MAFLD and CKD^30,31^, and obesity is known to have the potential to cause CKD due to the secretion of adipokines that increase hepatic insulin resistance and chronic inflammation^28^. In the present study, subjects in the MAFLD-only group had higher BMI levels, metabolic comorbidities (prediabetes, diabetes, and hypertension), NFS, and steatosis severity than the NAFLD-only group, consistent with recent studies in the United States^7,8,11^.

We also demonstrated that viral hepatitis with FLD further increased CKD risk. The hazard ratio was higher in MAFLD subjects with viral hepatitis than non-FLD subjects with viral hepatitis, either with or without MD (Supplementary Table S2). Although the risk of CKD in MAFLD with viral hepatitis was not significantly increased in a previous study, the previous study included a limited number of MAFLD subjects with HBV infection (*n* = 136)^10^. Another previous study did not explore the risk of incident CKD in the MAFLD population with viral hepatitis^18^. Accumulating evidence indicates that concomitant FLD and viral hepatitis have synergistic effects on more advanced hepatic fibrosis^5^, cardiovascular outcomes^32^, and mortality^5^.

The results of the present study indicate that FLD and metabolic dysregulation may have additive or synergistic effects on the occurrence of metabolic complications in those with excessive alcohol intake. Although the increase in the risk of CKD in subjects with excessive alcohol intake alone did not reach statistical significance, excessive alcohol intake in those with MD significantly increased the risk of CKD, and more prominently so in those with both MD and FLD, with significant interaction effects among excessive alcohol intake, MD, and FLD on the risk of CKD (Supplementary Table S1). Although the risk of CKD in MAFLD with excessive alcohol consumption was not found to be significantly increased in a previous study, the previous study might have had insufficient power due to the small size of this specific population in that study (*n* = 285)^10^. There was no interaction between the risk of CKD in fatty liver index-defined MAFLD and the presence of excessive alcohol consumption in another previous study^18^. However, it has been shown that alcohol and metabolic syndrome synergistically increase the risk of liver injury and liver disease progression^4^, although the contribution of such an effect to the risk of CKD remains unclear.

Our study has several strengths; it was a longitudinal study with a relatively large sample size with information available for multiple metabolic parameters in addition to detailed clinical data such as daily alcohol consumption, underlying diseases, and physical activities. In addition, all participants underwent abdominal ultrasounds, and experienced sonographers measured the severity of steatosis. Also, fibrosis risk, which is known to be associated with CKD^16,27^, was assessed by NFS and used as a covariate in the multivariable analyses.

Several limitations of this study should also be addressed. First, since this study was conducted in a healthy Korean population, our results may not be generalizable to other ethnicities or populations. Second, hepatic steatosis was not diagnosed using liver biopsy, although ultrasound is widely accessible and is the first-choice imaging modality for fatty liver screening in clinical and population settings^15,16^. Since alcohol intake was assessed via questionnaires, actual alcohol consumption may be higher than reported and the NAFLD population might be smaller than estimated. In this context, we utilized an Asian-specific threshold for excessive alcohol consumption (men > 20 g/day, women > 10 g/day) to minimize the likelihood of overestimation in the NAFLD group.

In conclusion, by using MAFLD criteria rather than NAFLD criteria, a greater number of individuals with an increased risk of developing CKD can be identified. The risk of developing CKD was not elevated in those with NAFLD who did not meet the MAFLD criteria or did not have overweight/obesity. However, there was a significant association between MAFLD patients who consumed excessive alcohol or had viral hepatitis and CKD, suggesting that an elevated risk of CKD associated with FLD should not be overlooked in these populations.

## Supplementary Information

Supplementary Information.

## Data Availability

The datasets generated and/or analyzed during this study are available from the corresponding authors on reasonable request.
