# Supplementary material for: MAFLD and NAFLD in the prediction of incident chronic kidney disease
Source: Sci Rep. 2023 Jan 31;13:1796. doi: 10.1038/s41598-023-27762-6 (PMC9889784; doi:10.1038/s41598-023-27762-6)
Supplement: Supplementary file 1 — Supplementary Information. [file 41598_2023_27762_MOESM1_ESM.docx]

**Supplementary Table S1** Associations of alcohol consumption, fatty liver disease, and metabolic dysregulation with incident CKD

|  | **Cases, *n*** | **Event, *n*** |  | **Hazard ratio (95% CI)** | | | | | | ***P for interaction^*^*** |
| --- | --- | --- | --- | --- | --- | --- | --- | --- | --- | --- |
|  |  |  | **Model 1**^†^ **(95%CI)** | | ***p*** | **Model 2^‡^ (95% CI)** | ***p*** | **Model 3^§^ (95%CI) *p*** | |  |
| Non-FLD without excessive alcohol intake^¶^ or MD^#^ | 8,211 | 226 | Ref | |  | Ref |  | Ref |  | N/A |
| Non-FLD with excessive alcohol^¶^ intake, without MD^#^ | 1,203 | 33 | 1.11 (0.77-1.60) | | 0.58 | 1.31 (0.90-1.91) | 0.19 | 1.37 (0.94-2.01) | 0.10 | N/A |
| Non-FLD with excessive alcohol intake^¶^, with MD^#^ | 1,260 | 55 | 1.74 (1.30-2.34) | | <0.01 | 1.83 (1.32-2.53) | <0.01 | 1.78 (1.28-2.48) | <0.01 | <0.01 |
| FLD with excessive alcohol intake^¶^, with MD^#^ | 1,576 | 111 | 2.83 (2.26-3.56) | | <0.01 | 3.21 (2.45-4.19) | <0.01 | 3.10 (2.34-4.10) | <0.01 | <0.01 |

^†^Model 1 was crude.

**^‡^**Model 2 was adjusted for age and sex.

**^§^**Model 3 was adjusted for age, sex, estimated glomerular filtration rate, smoking, physical activity, prediabetes, diabetes, hypertension, cardiovascular disease, NAFLD fibrosis score, body mass index.

^*^vs. Non-FLD with excessive alcohol^¶^ intake, without MD group

^¶^Excess consumption of alcohol was defined as more than 20 g daily of alcohol consumption in men and more than 10 g in women.

^#^Metabolic dysregulation; subjects with overweight/obese (body mass index ≥ 23 kg/m^2^) or had diabetes (fasting plasma glucose [FPG] ≥126 mg/dl, hemoglobin A1c [HbA1c] ≥ 6.5%, or specific drug treatment), or at least two of the following metabolic risk abnormalities: (i) waist circumference ≥ 90 cm in men and ≥80 cm in women; (ii) blood pressure ≥130/85 mmHg or specific drug treatment; (iii) triglycerides ≥150 mg/dl; (iv) HDL-C <40 mg/dl for men and <50 mg/dl for women; (v) prediabetes (FPG 100–125 mg/dl or HbA1c 5.7–6.4%); (vi) homeostasis model assessment of insulin resistance ≥2.5; and (vii) high-sensitivity C-reactive protein >2 mg/dl.

Abbreviations: CI, confidence interval; CKD, chronic kidney disease; FLD, fatty liver disease; MD, metabolic dysregulation; N/A, not appicable

**Supplementary Table S2** Associations of viral hepatitis, fatty liver disease, and metabolic dysregulation with incident CKD

|  | **Cases, *n*** | **Event, *n*** | **Hazard ratio (95% CI)** | | | | | | ***P for***  ***interaction^*^*** |
| --- | --- | --- | --- | --- | --- | --- | --- | --- | --- |
|  |  |  | **Model 1**^†^ **(95%CI)** | ***p*** | **Model 2^‡^ (95% CI)** | ***p*** | **Model 3^§^ (95%CI)** | ***p*** |  |
| Non-FLD without viral hepatitis or MD^¶^ | 8,989 | 245 | Ref |  | Ref |  | Ref |  | N/A |
| Non-FLD with viral hepatitis, without MD^¶^ | 425 | 14 | 1.19 (0.69-2.04) | 0.53 | 1.20 (0.70-2.05) | 0.51 | 1.06 (0.59-1.89) | 0.85 | N/A |
| Non-FLD with viral hepatitis, with MD^¶^ | 278 | 16 | 2.12 (1.28-3.52) | <0.01 | 1.99 (1.19-3.32) | <0.01 | 2.16 (1.26-3.71) | <0.01 | <0.01 |
| FLD with viral hepatitis, with MD^¶^ | 263 | 19 | 2.64 (1.66-4.22) | <0.01 | 2.68 (1.66-4.34) | <0.01 | 3.10 (1.81-5.29) | <0.01 | <0.01 |

^†^Model 1 was crude.

**^‡^**Model 2 was adjusted for age and sex.

**^§^**Model 3 was adjusted for age, sex, estimated glomerular filtration rate, smoking, physical activity, prediabetes, diabetes, hypertension, cardiovascular disease, NAFLD fibrosis score, body mass index, excessive alcohol consumption.

^*^vs. Non-FLD with viral hepatitis, without MD group

^¶^Metabolic dysregulation; subjects with overweight/obese (body mass index ≥ 23 kg/m^2^) or had diabetes (fasting plasma glucose [FPG] ≥126 mg/dl, hemoglobin A1c [HbA1c] ≥ 6.5%, or specific drug treatment), or at least two of the following metabolic risk abnormalities: (i) waist circumference ≥ 90 cm in men and ≥80 cm in women; (ii) blood pressure ≥130/85 mmHg or specific drug treatment; (iii) triglycerides ≥150 mg/dl; (iv) HDL-C <40mg/dl for men and <50mg/dl for women; (v) prediabetes (FPG 100–125 mg/dl or HbA1c 5.7–6.4%); (vi) homeostasis model assessment of insulin resistance ≥2.5; and (vii) high-sensitivity C-reactive protein >2 mg/dl.

Abbreviations: CI, confidence interval; CKD, chronic kidney disease; FLD, fatty liver disease; MD, metabolic dysregulation.
